# (Furfuryl­amino)­triphenyl­phospho­nium bromide

**DOI:** 10.1107/S1600536812050805

**Published:** 2012-12-22

**Authors:** Carla Martínez de León, Hugo Tlahuext, Angelina Flores-Parra, Angélica María Duarte-Hernández, Jean-Michel Grévy

**Affiliations:** a Centro de Investigaciones Químicas, Universidad Autónoma del Estado de Morelos. Av. Universidad 1001 Col., Chamilpa, CP 62100, Cuernavaca Mor., Mexico; b Departamento de Química, Cinvestav México, 07000 Mexico DF, Mexico

## Abstract

In the title salt, C_23_H_21_NOP^+^·Br^−^, the dihedral angles between the phenyl rings are 70.41 (18), 73.6 (2) and 80.85 (19)°. In the crystal, neighboring mol­ecules are linked through an N—H⋯Br hydrogen bond and four weak C—H⋯Br contacts, forming a three-dimensional network.

## Related literature

For (amino)­phospho­nium bromides derived from primary amines, see: Cao *et al.* (2010 ▶); Boubekeur *et al.* (2006 ▶); Dyer *et al.* (2011 ▶); Horner & Oediger (1959 ▶). For C—H⋯*X* hydrogen bonds, see: Jeffrey (1997 ▶); Zhang *et al.* (2003 ▶). For graph-set motifs, see: Bernstein, *et al.* (1995 ▶).
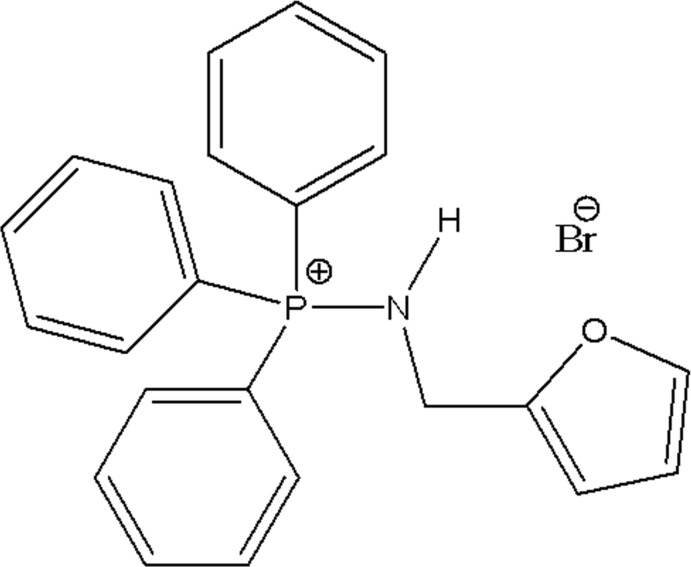


## Experimental

### 

#### Crystal data

C_23_H_21_NOP^+^·Br^−^
*M* *_r_* = 438.29
Triclinic, 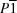

*a* = 9.5190 (19) Å
*b* = 9.812 (2) Å
*c* = 12.726 (3) Å
α = 110.30 (3)°
β = 104.89 (3)°
γ = 96.81 (3)°
*V* = 1048.7 (4) Å^3^
*Z* = 2
Mo *K*α radiation
μ = 2.05 mm^−1^
*T* = 293 K
0.20 × 0.17 × 0.13 mm

#### Data collection

Siemens P4 diffractometer
Absorption correction: multi-scan (*SADABS*; Sheldrick, 1996 ▶) *T*_min_ = 0.685, *T*_max_ = 0.777
11498 measured reflections
3673 independent reflections
3213 reflections with *I* > 2σ(*I*)
*R*_int_ = 0.046

#### Refinement

*R*[*F* ^2^ > 2σ(*F* ^2^)] = 0.038
*wR*(*F* ^2^) = 0.129
*S* = 1.02
3673 reflections
247 parameters
1 restraint
H atoms treated by a mixture of independent and constrained refinement
Δρ_max_ = 0.32 e Å^−3^
Δρ_min_ = −0.46 e Å^−3^

### 

Data collection: *XSCANS* (Siemens, 1994 ▶); cell refinement: *XSCANS*; data reduction: *SAINT-Plus NT* (Bruker, 2001 ▶); program(s) used to solve structure: *SHELXS97* (Sheldrick, 2008 ▶); program(s) used to refine structure: *SHELXL97* (Sheldrick, 2008 ▶); molecular graphics: *SHELXTL* (Sheldrick, 2008 ▶); software used to prepare material for publication: *PLATON* (Spek, 2009 ▶), *publCIF* (Westrip, 2010 ▶) and *DIAMOND* (Brandenburg, 2006 ▶).

## Supplementary Material

Crystal structure: contains datablock(s) I, global. DOI: 10.1107/S1600536812050805/is5227sup1.cif

Structure factors: contains datablock(s) I. DOI: 10.1107/S1600536812050805/is5227Isup2.hkl

Supplementary material file. DOI: 10.1107/S1600536812050805/is5227Isup3.cml

Additional supplementary materials:  crystallographic information; 3D view; checkCIF report

## Figures and Tables

**Table 1:** Hydrogen-bond geometry (Å, °)

*D*—H⋯*A*	*D*—H	H⋯*A*	*D*⋯*A*	*D*—H⋯*A*
N1—H1*A*⋯Br1	0.81 (3)	2.49 (3)	3.293 (3)	170 (4)
C8—H8⋯Br1^i^	0.93	2.98	3.835 (4)	153
C15—H15⋯Br1^ii^	0.93	3.00	3.728 (4)	137
C21—H21⋯Br1^iii^	0.93	2.94	3.829 (6)	161
C23—H23⋯Br1^iv^	0.93	2.97	3.782 (4)	147

Symmetry codes: (i) 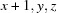; (ii) 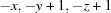; (iii) 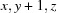; (iv) 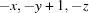.

**Fig. 1.**
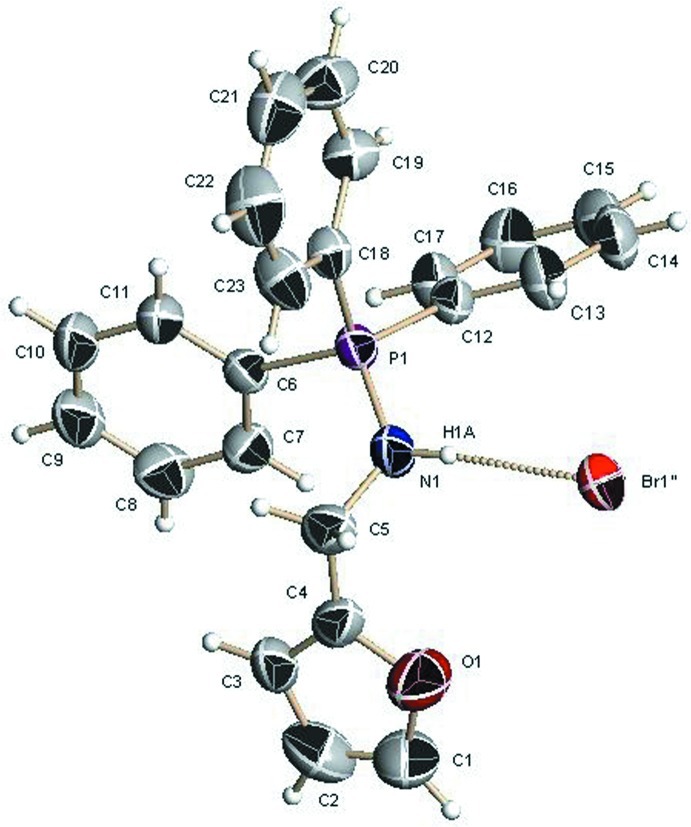
The molecular structure of the title compound, showing the atom-labelling scheme. Displacement ellipsoids are drawn at the 50% probability level and H atoms are shown as small spheres of arbitrary radius.

**Fig. 2.**
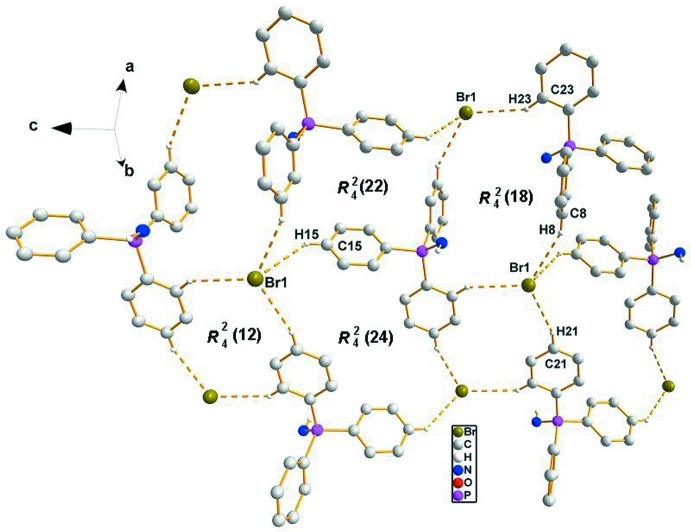
A view of the C—H···Br interactions (dashed lines), showing the R24(12), R24(18), R24(22) and R24(24) graph set motifs. The furfuryl group and hydrogen atoms not involved in hydrogen bonding have been omitted for clarity.

## supplementary crystallographic information

### Comment

Aminophosphonium salts are important species because of their reactivity and their role as intermediates in numerous organophosphorus reactions. For instance, aminophosphonium derived from primary amine are key intermediates in the preparation of iminophosphoranes through the Horner-Oediger reaction (Horner & Oediger, 1959). Apart from their use in the aza-Wittig reaction, this class of compounds became very important ligands during the past few years due to their good activity in various catalytic processes when coordinated to transition metals (Dyer *et al.*, 2011). Here we report the structure of the (furfurylamino) triphenylphosphonium bromide obtained as an intermediate during the synthesis of the corresponding iminophosphorane.

In the crystal structure, neighboring molecules are linked through an N—H···Br hydrogen bond and four weak C—H···Br contacts (Jeffrey, 1997; Zhang, *et al.*, 2003; Table 1), forming a three-dimensional network. In the hydrogen-bond pattern, the C—H···Br contacts form corrugated sheets. These sheets are composed of *R*^2^_4_(12), *R*^2^_4_(18), *R*^2^_4_(22) and *R*^2^_4_(24) graph set motifs (Bernstein, *et al.* 1995; Fig. 2). Neighboring sheets are further linked by N—H···Br hydrogen bonds, generating the three-dimensional network.

### Experimental

A mixture of 630 mg of DABCO (5.65 mmol) and 1 ml of furfurylamine (11.31 mmol) in 10 ml of dry benzene was added dropwise through canula between 0 and 5 °C to a stirred suspension of Ph_3_PBr_2_ (11.31 mmol) in 20 ml of dry benzene. After 3 h of stirring at ambient temperature, 10 ml of distilled water were added to the medium and the compound extracted with 20 ml of methylene chloride. The organic phase was further washed with 10 ml of water, dried over MgSO_4_ and all volatiles were eliminated under vacuum. The off white powder obtained was suspended in Et_2_O and left under stirring overnight. After filtration of the suspension, the solid was crystallized from hot THF giving 4.05 g (81%) of colorless crystals of the title compound, which were suitable for X-ray crystal structure analysis and fully characterized by standard analytical methods. ^31^P NMR (CH_2_Cl_2_) 30.03 p.p.m.; m. p. 420 K.

### Refinement

H atoms were positioned geometrically and constrained using the riding-model approximation [C—H_aryl_ = 0.93 Å, *U*_iso_(H_aryl_)= 1.2 *U*_eq_(C); C—H_methylene_ = 0.97 Å, *U*_iso_(H_methylene_) = 1.2*U*_eq_(C)]. The hydrogen atom bonded to N1 was located in a difference Fourier map. Its coordinates were refined with a distance restraint N—H = 0.86 Å, and with *U*_iso_(H) = 1.2*U*_eq_(N).

### Crystal data

**Table:**

C_23_H_21_NOP^+^·Br^−^	*Z* = 2
*M**_r_* = 438.29	*F*(000) = 448
Triclinic, *P*1	*D*_x_ = 1.388 Mg m^−^^3^
Hall symbol: -P 1	Melting point: 420 K
*a* = 9.5190 (19) Å	Mo *K*α radiation, λ = 0.71073 Å
*b* = 9.812 (2) Å	Cell parameters from 3600 reflections
*c* = 12.726 (3) Å	θ = 4.2–30°
α = 110.30 (3)°	µ = 2.05 mm^−^^1^
β = 104.89 (3)°	*T* = 293 K
γ = 96.81 (3)°	Plate, colourless
*V* = 1048.7 (4) Å^3^	0.20 × 0.17 × 0.13 mm

### Data collection

**Table:**

Siemens P4 diffractometer	3673 independent reflections
Radiation source: fine-focus sealed tube	3213 reflections with *I* > 2σ(*I*)
Graphite monochromator	*R*_int_ = 0.046
Detector resolution: 8.3 pixels mm^-1^	θ_max_ = 25.0°, θ_min_ = 4.2°
ω scans	*h* = −11→11
Absorption correction: multi-scan (*SADABS*; Sheldrick, 1996)	*k* = −11→11
*T*_min_ = 0.685, *T*_max_ = 0.777	*l* = −15→15
11498 measured reflections	

### Refinement

**Table:**

Refinement on *F*^2^	Primary atom site location: structure-invariant direct methods
Least-squares matrix: full	Secondary atom site location: difference Fourier map
*R*[*F*^2^ > 2σ(*F*^2^)] = 0.038	Hydrogen site location: inferred from neighbouring sites
*wR*(*F*^2^) = 0.129	H atoms treated by a mixture of independent and constrained refinement
*S* = 1.02	*w* = 1/[σ^2^(*F*_o_^2^) + (0.1*P*)^2^ + 0.001*P*] where *P* = (*F*_o_^2^ + 2*F*_c_^2^)/3
3673 reflections	(Δ/σ)_max_ = 0.001
247 parameters	Δρ_max_ = 0.32 e Å^−^^3^
1 restraint	Δρ_min_ = −0.46 e Å^−^^3^

### Special details

**Table:**

Geometry. All e.s.d.'s (except the e.s.d. in the dihedral angle between two l.s. planes) are estimated using the full covariance matrix. The cell e.s.d.'s are taken into account individually in the estimation of e.s.d.'s in distances, angles and torsion angles; correlations between e.s.d.'s in cell parameters are only used when they are defined by crystal symmetry. An approximate (isotropic) treatment of cell e.s.d.'s is used for estimating e.s.d.'s involving l.s. planes.
Refinement. Refinement of *F*^2^ against ALL reflections. The weighted *R*-factor *wR* and goodness of fit *S* are based on *F*^2^, conventional *R*-factors *R* are based on *F*, with *F* set to zero for negative *F*^2^. The threshold expression of *F*^2^ > σ(*F*^2^) is used only for calculating *R*-factors(gt) *etc*. and is not relevant to the choice of reflections for refinement. *R*-factors based on *F*^2^ are statistically about twice as large as those based on *F*, and *R*- factors based on ALL data will be even larger.

### Fractional atomic coordinates and isotropic or equivalent isotropic displacement parameters (Å^2^)

**Table:**

	*x*	*y*	*z*	*U*_iso_*/*U*_eq_	
Br1	−0.09719 (3)	0.34393 (3)	0.15232 (3)	0.05548 (17)	
C1	0.3290 (6)	0.1167 (5)	0.0155 (5)	0.0990 (16)	
H1	0.3038	0.0160	0.0015	0.119*	
C2	0.4595 (5)	0.1890 (6)	0.0228 (4)	0.0888 (13)	
H2	0.5425	0.1495	0.0163	0.107*	
C3	0.4491 (4)	0.3371 (4)	0.0425 (3)	0.0671 (9)	
H3	0.5230	0.4131	0.0485	0.081*	
C4	0.3156 (4)	0.3483 (3)	0.0509 (3)	0.0562 (8)	
C5	0.2489 (4)	0.4800 (4)	0.0737 (3)	0.0582 (8)	
H5A	0.3108	0.5592	0.0654	0.070*	
H5B	0.1508	0.4539	0.0157	0.070*	
C6	0.4994 (3)	0.7451 (3)	0.2917 (2)	0.0435 (6)	
C7	0.5865 (4)	0.6410 (3)	0.2895 (3)	0.0564 (7)	
H7	0.5496	0.5515	0.2936	0.068*	
C8	0.7281 (4)	0.6699 (4)	0.2812 (4)	0.0693 (9)	
H8	0.7861	0.5996	0.2788	0.083*	
C9	0.7826 (4)	0.8027 (4)	0.2765 (3)	0.0690 (10)	
H9	0.8779	0.8225	0.2711	0.083*	
C10	0.6976 (4)	0.9062 (4)	0.2797 (3)	0.0651 (9)	
H10	0.7360	0.9963	0.2771	0.078*	
C11	0.5550 (4)	0.8782 (3)	0.2867 (3)	0.0523 (7)	
H11	0.4973	0.9485	0.2880	0.063*	
C12	0.3181 (3)	0.6870 (3)	0.4351 (3)	0.0447 (6)	
C13	0.1854 (4)	0.6505 (4)	0.4558 (3)	0.0623 (8)	
H13	0.0946	0.6267	0.3965	0.075*	
C14	0.1884 (4)	0.6497 (4)	0.5642 (3)	0.0690 (9)	
H14	0.0992	0.6258	0.5781	0.083*	
C15	0.3221 (4)	0.6841 (4)	0.6525 (3)	0.0638 (9)	
H15	0.3228	0.6832	0.7255	0.077*	
C16	0.4526 (4)	0.7190 (4)	0.6332 (3)	0.0659 (9)	
H16	0.5427	0.7420	0.6930	0.079*	
C17	0.4520 (4)	0.7205 (4)	0.5243 (3)	0.0545 (7)	
H17	0.5418	0.7440	0.5113	0.065*	
C18	0.2227 (3)	0.8445 (3)	0.2856 (2)	0.0443 (6)	
C19	0.2166 (4)	0.9579 (4)	0.3855 (3)	0.0545 (7)	
H19	0.2557	0.9558	0.4597	0.065*	
C20	0.1531 (4)	1.0735 (4)	0.3751 (4)	0.0727 (10)	
H20	0.1491	1.1490	0.4423	0.087*	
C21	0.0956 (4)	1.0780 (5)	0.2661 (5)	0.0790 (12)	
H21	0.0534	1.1565	0.2591	0.095*	
C22	0.1011 (5)	0.9652 (6)	0.1673 (4)	0.0837 (12)	
H22	0.0630	0.9685	0.0935	0.100*	
C23	0.1620 (4)	0.8474 (4)	0.1758 (3)	0.0660 (9)	
H23	0.1621	0.7703	0.1080	0.079*	
H1A	0.157 (3)	0.490 (4)	0.193 (4)	0.079*	
N1	0.2354 (3)	0.5341 (3)	0.1936 (2)	0.0576 (7)	
O1	0.2369 (3)	0.2133 (3)	0.0317 (3)	0.0935 (9)	
P1	0.31407 (8)	0.69765 (8)	0.29668 (6)	0.0415 (2)	

### Atomic displacement parameters (Å^2^)

**Table:**

	*U*^11^	*U*^22^	*U*^33^	*U*^12^	*U*^13^	*U*^23^
Br1	0.0476 (2)	0.0683 (2)	0.0579 (2)	0.01512 (15)	0.02311 (15)	0.02845 (17)
C1	0.118 (4)	0.070 (2)	0.147 (4)	0.049 (3)	0.080 (4)	0.049 (3)
C2	0.078 (3)	0.106 (3)	0.085 (3)	0.041 (3)	0.038 (2)	0.026 (2)
C3	0.058 (2)	0.064 (2)	0.068 (2)	−0.0066 (16)	0.0343 (16)	0.0094 (16)
C4	0.071 (2)	0.0468 (15)	0.0448 (16)	0.0052 (14)	0.0174 (14)	0.0147 (13)
C5	0.071 (2)	0.0541 (17)	0.0449 (16)	0.0118 (15)	0.0177 (14)	0.0151 (13)
C6	0.0419 (14)	0.0445 (14)	0.0417 (14)	0.0072 (11)	0.0166 (11)	0.0128 (11)
C7	0.0508 (17)	0.0490 (15)	0.072 (2)	0.0138 (13)	0.0261 (14)	0.0223 (14)
C8	0.0502 (18)	0.069 (2)	0.088 (2)	0.0212 (16)	0.0285 (17)	0.0226 (19)
C9	0.0449 (17)	0.075 (2)	0.075 (2)	−0.0001 (16)	0.0280 (16)	0.0140 (18)
C10	0.058 (2)	0.0620 (19)	0.070 (2)	−0.0046 (15)	0.0287 (17)	0.0207 (17)
C11	0.0536 (17)	0.0487 (15)	0.0551 (17)	0.0071 (13)	0.0227 (14)	0.0187 (13)
C12	0.0485 (16)	0.0439 (14)	0.0486 (15)	0.0119 (11)	0.0203 (12)	0.0223 (12)
C13	0.0513 (18)	0.085 (2)	0.0552 (18)	0.0065 (16)	0.0207 (14)	0.0328 (17)
C14	0.069 (2)	0.082 (2)	0.071 (2)	0.0107 (18)	0.0389 (18)	0.0381 (19)
C15	0.084 (2)	0.0650 (19)	0.0555 (18)	0.0161 (17)	0.0283 (17)	0.0353 (16)
C16	0.064 (2)	0.079 (2)	0.058 (2)	0.0146 (17)	0.0115 (16)	0.0368 (18)
C17	0.0498 (17)	0.0644 (18)	0.0545 (17)	0.0096 (14)	0.0173 (13)	0.0303 (15)
C18	0.0375 (14)	0.0532 (15)	0.0461 (15)	0.0113 (12)	0.0138 (11)	0.0234 (13)
C19	0.0508 (17)	0.0547 (16)	0.0538 (17)	0.0143 (13)	0.0115 (13)	0.0196 (14)
C20	0.061 (2)	0.0589 (19)	0.092 (3)	0.0228 (16)	0.0202 (19)	0.0232 (19)
C21	0.058 (2)	0.080 (2)	0.117 (4)	0.0233 (19)	0.022 (2)	0.063 (3)
C22	0.074 (3)	0.121 (3)	0.086 (3)	0.034 (2)	0.023 (2)	0.075 (3)
C23	0.064 (2)	0.092 (2)	0.0545 (19)	0.0258 (18)	0.0226 (16)	0.0379 (18)
N1	0.0556 (16)	0.0542 (14)	0.0539 (15)	−0.0049 (12)	0.0266 (12)	0.0099 (12)
O1	0.0757 (18)	0.0658 (15)	0.141 (3)	0.0168 (13)	0.0499 (18)	0.0326 (17)
P1	0.0393 (4)	0.0451 (4)	0.0424 (4)	0.0081 (3)	0.0177 (3)	0.0171 (3)

### Geometric parameters (Å, º)

**Table:**

Br1—H1A	2.49 (2)	C12—C13	1.387 (4)
C1—C2	1.319 (7)	C12—P1	1.791 (3)
C1—O1	1.365 (5)	C13—C14	1.376 (5)
C1—H1	0.9300	C13—H13	0.9300
C2—C3	1.407 (6)	C14—C15	1.377 (6)
C2—H2	0.9300	C14—H14	0.9300
C3—C4	1.317 (5)	C15—C16	1.354 (5)
C3—H3	0.9300	C15—H15	0.9300
C4—O1	1.350 (4)	C16—C17	1.390 (5)
C4—C5	1.479 (5)	C16—H16	0.9300
C5—N1	1.477 (4)	C17—H17	0.9300
C5—H5A	0.9700	C18—C23	1.380 (4)
C5—H5B	0.9700	C18—C19	1.389 (4)
C6—C11	1.379 (4)	C18—P1	1.796 (3)
C6—C7	1.387 (4)	C19—C20	1.377 (5)
C6—P1	1.794 (3)	C19—H19	0.9300
C7—C8	1.383 (5)	C20—C21	1.372 (6)
C7—H7	0.9300	C20—H20	0.9300
C8—C9	1.371 (6)	C21—C22	1.375 (7)
C8—H8	0.9300	C21—H21	0.9300
C9—C10	1.368 (6)	C22—C23	1.378 (6)
C9—H9	0.9300	C22—H22	0.9300
C10—C11	1.384 (5)	C23—H23	0.9300
C10—H10	0.9300	N1—P1	1.614 (3)
C11—H11	0.9300	N1—H1A	0.817 (19)
C12—C17	1.386 (4)		
			
C2—C1—O1	109.1 (4)	C12—C13—H13	120.1
C2—C1—H1	125.4	C13—C14—C15	120.7 (3)
O1—C1—H1	125.4	C13—C14—H14	119.7
C1—C2—C3	106.9 (4)	C15—C14—H14	119.7
C1—C2—H2	126.5	C16—C15—C14	120.2 (3)
C3—C2—H2	126.5	C16—C15—H15	119.9
C4—C3—C2	107.4 (3)	C14—C15—H15	119.9
C4—C3—H3	126.3	C15—C16—C17	120.1 (3)
C2—C3—H3	126.3	C15—C16—H16	120.0
C3—C4—O1	109.4 (3)	C17—C16—H16	120.0
C3—C4—C5	129.4 (3)	C12—C17—C16	120.3 (3)
O1—C4—C5	121.2 (3)	C12—C17—H17	119.9
N1—C5—C4	111.2 (3)	C16—C17—H17	119.9
N1—C5—H5A	109.4	C23—C18—C19	119.3 (3)
C4—C5—H5A	109.4	C23—C18—P1	119.2 (2)
N1—C5—H5B	109.4	C19—C18—P1	121.4 (2)
C4—C5—H5B	109.4	C20—C19—C18	120.2 (3)
H5A—C5—H5B	108.0	C20—C19—H19	119.9
C11—C6—C7	119.8 (3)	C18—C19—H19	119.9
C11—C6—P1	122.2 (2)	C21—C20—C19	120.4 (4)
C7—C6—P1	117.9 (2)	C21—C20—H20	119.8
C8—C7—C6	120.2 (3)	C19—C20—H20	119.8
C8—C7—H7	119.9	C20—C21—C22	119.3 (4)
C6—C7—H7	119.9	C20—C21—H21	120.4
C9—C8—C7	119.6 (3)	C22—C21—H21	120.4
C9—C8—H8	120.2	C21—C22—C23	121.2 (4)
C7—C8—H8	120.2	C21—C22—H22	119.4
C10—C9—C8	120.4 (3)	C23—C22—H22	119.4
C10—C9—H9	119.8	C22—C23—C18	119.5 (4)
C8—C9—H9	119.8	C22—C23—H23	120.2
C9—C10—C11	120.6 (3)	C18—C23—H23	120.2
C9—C10—H10	119.7	C5—N1—P1	125.8 (2)
C11—C10—H10	119.7	C5—N1—H1A	110 (3)
C6—C11—C10	119.4 (3)	P1—N1—H1A	119 (3)
C6—C11—H11	120.3	C4—O1—C1	107.1 (3)
C10—C11—H11	120.3	N1—P1—C12	107.89 (14)
C17—C12—C13	119.0 (3)	N1—P1—C6	107.03 (14)
C17—C12—P1	121.1 (2)	C12—P1—C6	111.12 (14)
C13—C12—P1	119.9 (2)	N1—P1—C18	115.68 (15)
C14—C13—C12	119.8 (3)	C12—P1—C18	106.98 (13)
C14—C13—H13	120.1	C6—P1—C18	108.18 (13)
			
O1—C1—C2—C3	−1.0 (6)	C19—C18—C23—C22	2.2 (5)
C1—C2—C3—C4	2.6 (5)	P1—C18—C23—C22	−175.4 (3)
C2—C3—C4—O1	−3.1 (5)	C4—C5—N1—P1	119.5 (3)
C2—C3—C4—C5	178.7 (4)	C3—C4—O1—C1	2.5 (5)
C3—C4—C5—N1	−108.5 (4)	C5—C4—O1—C1	−179.1 (4)
O1—C4—C5—N1	73.5 (4)	C2—C1—O1—C4	−0.8 (6)
C11—C6—C7—C8	−0.6 (5)	C5—N1—P1—C12	−160.2 (3)
P1—C6—C7—C8	177.6 (3)	C5—N1—P1—C6	−40.5 (3)
C6—C7—C8—C9	0.7 (6)	C5—N1—P1—C18	80.1 (3)
C7—C8—C9—C10	−0.2 (6)	C17—C12—P1—N1	120.1 (3)
C8—C9—C10—C11	−0.5 (6)	C13—C12—P1—N1	−62.5 (3)
C7—C6—C11—C10	−0.1 (5)	C17—C12—P1—C6	3.1 (3)
P1—C6—C11—C10	−178.2 (2)	C13—C12—P1—C6	−179.5 (2)
C9—C10—C11—C6	0.7 (5)	C17—C12—P1—C18	−114.8 (3)
C17—C12—C13—C14	0.7 (5)	C13—C12—P1—C18	62.6 (3)
P1—C12—C13—C14	−176.7 (3)	C11—C6—P1—N1	128.4 (3)
C12—C13—C14—C15	−0.4 (6)	C7—C6—P1—N1	−49.7 (3)
C13—C14—C15—C16	0.0 (6)	C11—C6—P1—C12	−114.0 (3)
C14—C15—C16—C17	0.1 (6)	C7—C6—P1—C12	67.8 (3)
C13—C12—C17—C16	−0.7 (5)	C11—C6—P1—C18	3.2 (3)
P1—C12—C17—C16	176.7 (3)	C7—C6—P1—C18	−175.0 (2)
C15—C16—C17—C12	0.3 (5)	C23—C18—P1—N1	−39.4 (3)
C23—C18—C19—C20	−1.2 (5)	C19—C18—P1—N1	143.0 (3)
P1—C18—C19—C20	176.4 (3)	C23—C18—P1—C12	−159.6 (3)
C18—C19—C20—C21	−0.2 (6)	C19—C18—P1—C12	22.8 (3)
C19—C20—C21—C22	0.5 (7)	C23—C18—P1—C6	80.6 (3)
C20—C21—C22—C23	0.6 (7)	C19—C18—P1—C6	−97.0 (3)
C21—C22—C23—C18	−2.0 (6)		

### Hydrogen-bond geometry (Å, º)

**Table:**

*D*—H···*A*	*D*—H	H···*A*	*D*···*A*	*D*—H···*A*
N1—H1*A*···Br1	0.81 (3)	2.49 (3)	3.293 (3)	170 (4)
C8—H8···Br1^i^	0.93	2.98	3.835 (4)	153
C15—H15···Br1^ii^	0.93	3.00	3.728 (4)	137
C21—H21···Br1^iii^	0.93	2.94	3.829 (6)	161
C23—H23···Br1^iv^	0.93	2.97	3.782 (4)	147

Symmetry codes: (i) *x*+1, *y*, *z*; (ii) −*x*, −*y*+1, −*z*+1; (iii) *x*, *y*+1, *z*; (iv) −*x*, −*y*+1, −*z*.
